# Mechanisms of action of triptolide against colorectal cancer: insights from proteomic and phosphoproteomic analyses

**DOI:** 10.18632/aging.203992

**Published:** 2022-04-02

**Authors:** Xinqiang Song, Huanhuan He, Yu Zhang, Jinke Fan, Lei Wang

**Affiliations:** 1 College of Life Sciences, Xinyang Normal University, Xinyang 464000, China; 2 College of Medicine, Xinyang Normal University, Xinyang 464000, China

**Keywords:** colorectal cancer, triptolide, proteomic, phosphoproteomic, molecular docking

## Abstract

Triptolide is a potent anti-inflammatory agent that also possesses anticancer activity, including against colorectal cancer (CRC), one of the most frequent cancers around the world. In order to clarify how triptolide may be effective against CRC, we analyzed the proteome and phosphoproteome of CRC cell line HCT116 after incubation for 48 h with the drug (40 nM) or vehicle. Tandem mass tagging led to the identification of 403 proteins whose levels increased and 559 whose levels decreased in the presence of triptolide. We also identified 3,110 sites in proteins that were phosphorylated at higher levels and 3,161 sites phosphorylated at lower levels in the presence of the drug. Analysis of these differentially expressed and/or phosphorylated proteins showed that they were enriched in pathways involving ribosome biogenesis, PI3K−Akt signaling, MAPK signaling, nucleic acid binding as well as other pathways. Protein–protein interactions were explored using the STRING database, and we identified nine protein modules and 15 hub proteins. Finally, we identified 57 motifs using motif analysis of phosphosites and found 16 motifs were experimentally verified for known protein kinases, while 41 appear to be novel. These findings may help clarify how triptolide works against CRC and may guide the development of novel treatments.

## INTRODUCTION

Colorectal cancer (CRC) is one of the most frequent cancers, with more than 1.2 million new cases and 500,00 deaths annually around the world, the cornerstones of therapy are surgery, radiotherapy (for patients with rectal cancer), and chemotherapy [1]. Triptolide, the major active component of *Triptergium wilfordii* Hook. f, works against CRC by inhibiting colon cancer cell proliferation, colony formation, and organoid growth *in vitro* [2, 3]. The triptolide analog minnelide markedly inhibits the growth of CRC xenografts and the metastasis of CRC to liver, more than doubling the median survival of animals whose CRC has metastasized to the liver [4]. Triptolide also appears to inhibit the epithelial-mesenchymal transition and growth of colon cancer stem cells [5]. Thus, triptolide shows strong potential to treat CRC, but how it works is controversial.

Here we explored protein expression and phosphorylation in CRC cells treated with triptolide in an effort to identify the molecules and pathways that may mediate the drug’s anticancer effects. We applied quantitative proteomics and phosphoproteomics based on tandem mass tagging and nanospray liquid chromatography-tandem mass spectrometry. Proteomics allows global analysis of complex changes in protein expression [6, 7], and tandem mass tagging allows high-throughput, high-resolution quantification of changes in protein levels and their phosphorylation [8–10]. Our analyses may help clarify the anticancer mechanism of triptolide and identify druggable targets.

## RESULTS

### Proteome and phosphoproteome in HCT-116 cells

**Figure 1:**
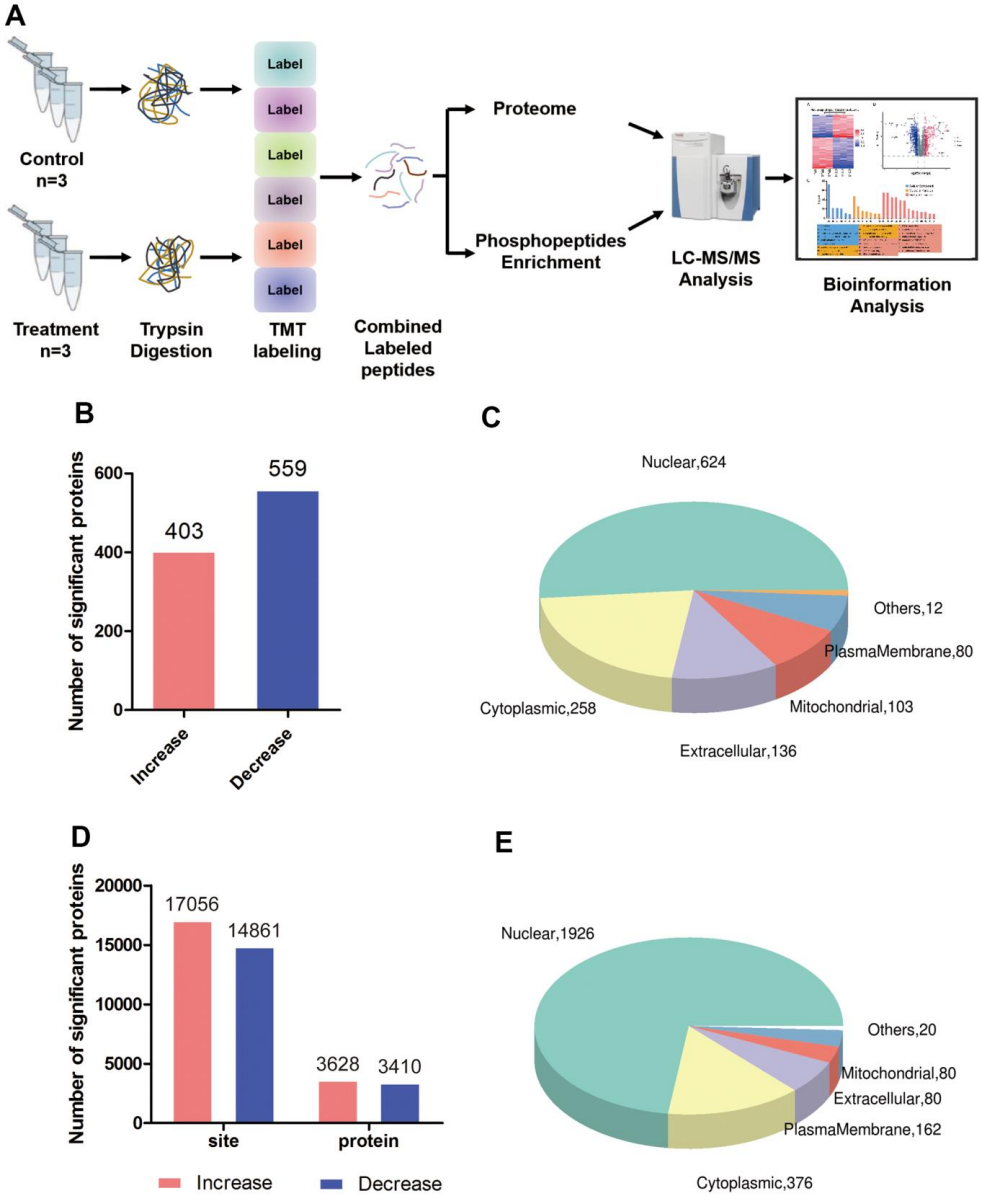
**Global proteomic and phosphoproteomic analysis of colorectal cancer cells.** (**A**) Schematic of the experimental workflow; LC, liquid chromatography; MS, mass spectrometry; TMT, tandem mass tags. (**B**) Numbers of proteins whose levels were significantly higher (red) or lower (blue) in triptolide-treated cell cultures than in control cultures. (**C**) Numbers of differentially expressed proteins in different subcellular compartments. (**D**) Numbers of sites in proteins whose phosphorylation was significantly higher (red) or lower (blue) in triptolide-treated cell cultures than in control cultures. (**E**) Numbers of differentially phosphorylated proteins in different subcellular compartments.

Using tandem mass tagging of total proteins as well as enrichment for phosphopeptides, followed by tandem mass spectrometry (Figure 1A), we identified 33,390 unique peptides corresponding to 5,860 proteins, of which 5,710 proteins could be quantified in triptolide-treated and control groups (Supplementary Table 1 and Supplementary Figures 1, 2A). Of these, 962 proteins were differentially expressed: 403 were present at higher levels and 559 proteins at lower levels in the presence of triptolide (Figure 1B). Triptolide was also associated with higher levels of phosphorylation at 3,110 sites in proteins and lower phosphorylation at 3,161 sites (Figure 1D). Most differentially expressed and/or phosphorylated proteins localized to the nucleus and cytoplasm (Figure 1C, 1E).

### Functional analysis of differentially expressed proteins in CRC

**Figure 2:**
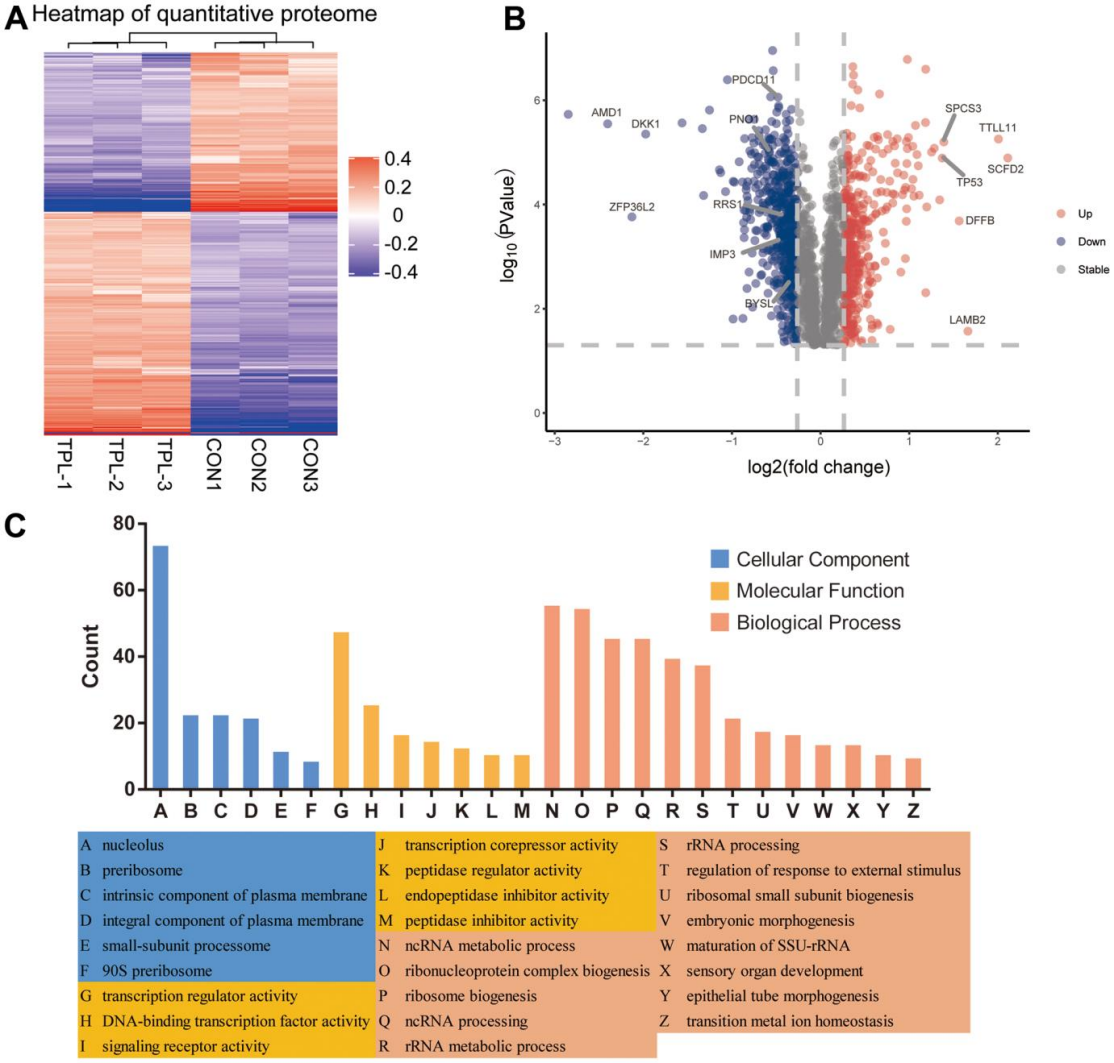
**Differential expression levels of the quantitative proteome and their enrichment in Gene Ontology terms.** (**A**) Heatmap of the quantitative proteome based on fold differences in expression. (**B**) Volcano plot of the differences in protein levels. The volcano map was drawn based on the expression of FC and P value (T-test). The significantly down-regulated proteins were blue (FC< 0.83 and P <0.05), the significantly up-regulated proteins were red (FC>1.2 and P <0.05), and the proteins with no difference were gray. (**C**) Classification of differentially expressed proteins based on Gene Ontology biological processes, cellular components and molecular functions.

A total of 5710 quantitative proteins were identified in the proteome analysis (Supplementary Figure 2A). We defined proteins that were significantly different (Student’s t-test, p < 0.05) and used the criterion of 1.2-fold or greater change as the criteria to screen candidate proteins, finally we identified 403 proteins with higher levels and 559 proteins with lower levels in the triptolide-treated group than in the control group (Figure 2A). Heatmaps were applied to indicate the expression levels of the differentially expressed proteins screened by the volcano map in three replicate samples of the triptolide-treated group and the control group (Figure 2B). The potential functions of these proteins were explored based on enrichment in GO terms (Figure 2C and Supplementary Figure 3A–3C). They were enriched in the following GO biological processes: rRNA processing, ribosome biogenesis, keratinocyte proliferation, maturation of SSU−rRNA, regulation of keratinocyte proliferation, RNA phosphodiester bond hydrolysis, and endonucleolysis. The differentially expressed proteins were enriched in the following GO cellular components: preribosome, small−subunit processome, 90S preribosome, MCM complex, intrinsic components of the plasma membrane, intrinsic components of the membrane, integral components of the plasma membrane, and nucleolus. The differentially expressed proteins were enriched in the following GO molecular functions: peptidase inhibitor activity, endopeptidase inhibitor activity, peptidase regulator activity, translation repressor activity, olfactory receptor activity, metalloendopeptidase inhibitor activity, transcription corepressor activity, signaling receptor activity and transmembrane signaling receptor activity.

### Analysis of differentially expressed proteins for enrichment in domains and KEGG pathways, protein-protein interactions and modules

**Figure 3:**
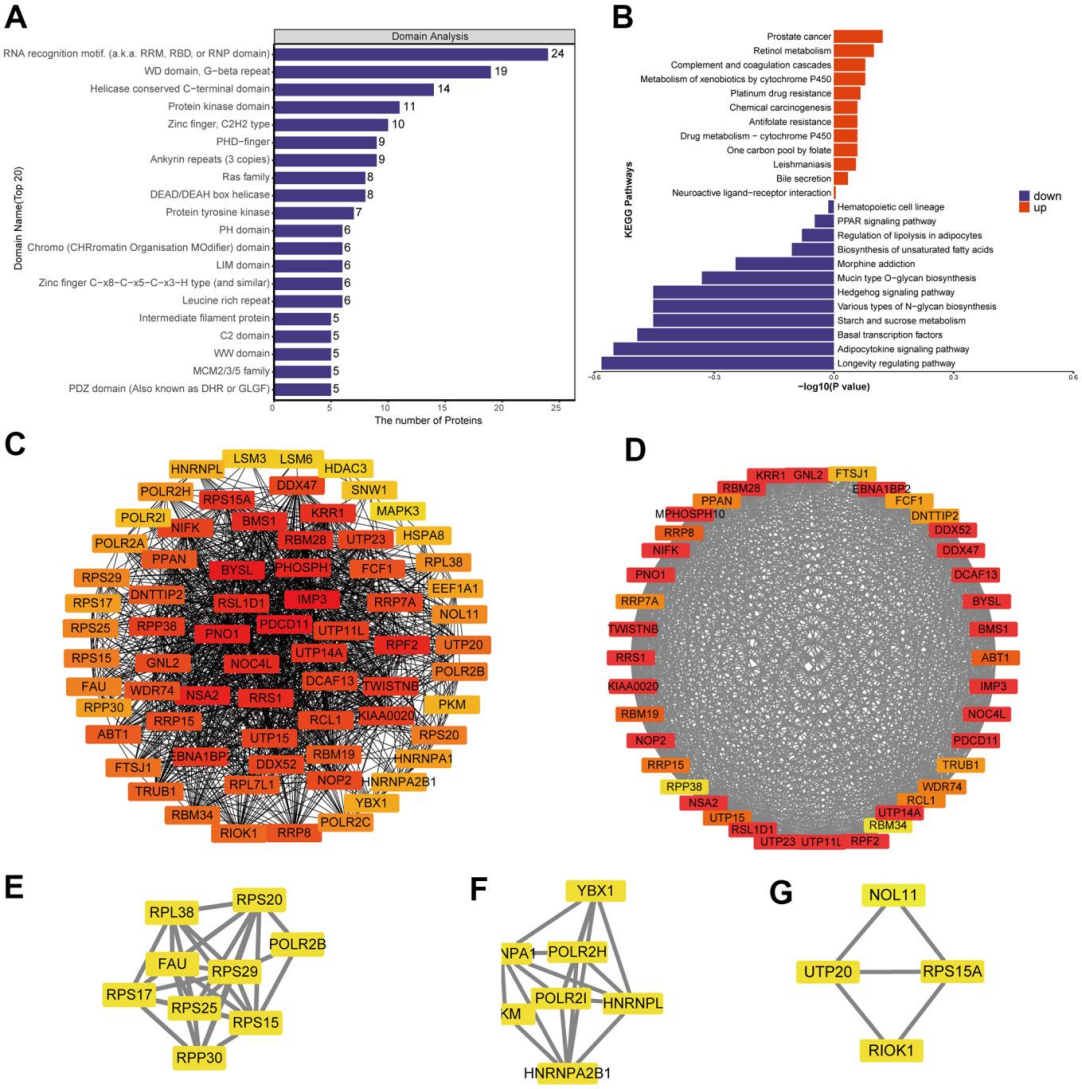
**Analysis of predicted interactions among differentially expressed proteins.** The four most significant modules were identified by the molecular complex detection (MCODE) algorithm. (**A**) Enrichment of domains in differentially expressed proteins. (**B**) Enrichment of KEGG pathways in differentially expressed proteins. (**C**) Interaction network of differentially expressed proteins. (**D**–**G**) The four most significant MCODE modules.

Differentially expressed proteins were enriched with the following domains (Figure 3A and Supplementary Figure 3E): PHD−finger, leucine-rich repeat, N−terminal MCM, CHRromatin Organisation MOdifier (“Chromo”), MCM2/3/5 family, MCM OB, and EGF−like. We identified several KEGG pathways that were enriched in upregulated proteins: chemical carcinogenesis, bile secretion, complement and coagulation cascades, prostate cancer and drug metabolism-cytochrome P450 (Figure 3B and Supplementary Figure 3D). Several KEGG pathways were enriched in downregulated proteins: PPAR signaling, mucin type O-glycan biosynthesis, starch and sucrose metabolism, various types of N-glycan biosynthesis, hedgehog signaling, basal transcription factors and longevity-regulating pathway.

We predicted interactions among differentially expressed proteins using STRING and Cytoscape (Figure 3C), and the protein-protein interaction network revealed four critical protein groups (Figure 3D–3G): MCODE 1 (MCODE score = 37.436), consisting of 40 nodes and 730 edges; MCODE 2 (score = 7.5), consisting of 9 nodes and 306 edges; MCODE 3 (score = 5.667), comprising 7 nodes and 17 edges; and MCODE 4 (score = 3.333), consisting of 4 nodes and 5 edges. Four classification methods in CytoHubba were used to identify the top 10 proteins (Supplementary Table 1), which when combined with the analysis of MCODE modules identified seven proteins as hub proteins: IMP3, BYSL, PDCD11, PNO1, NSA2, RRS1 and RPF2 (Supplementary Figure 6A).

### Functional analysis of differentially phosphorylated proteins in CRC

**Figure 4:**
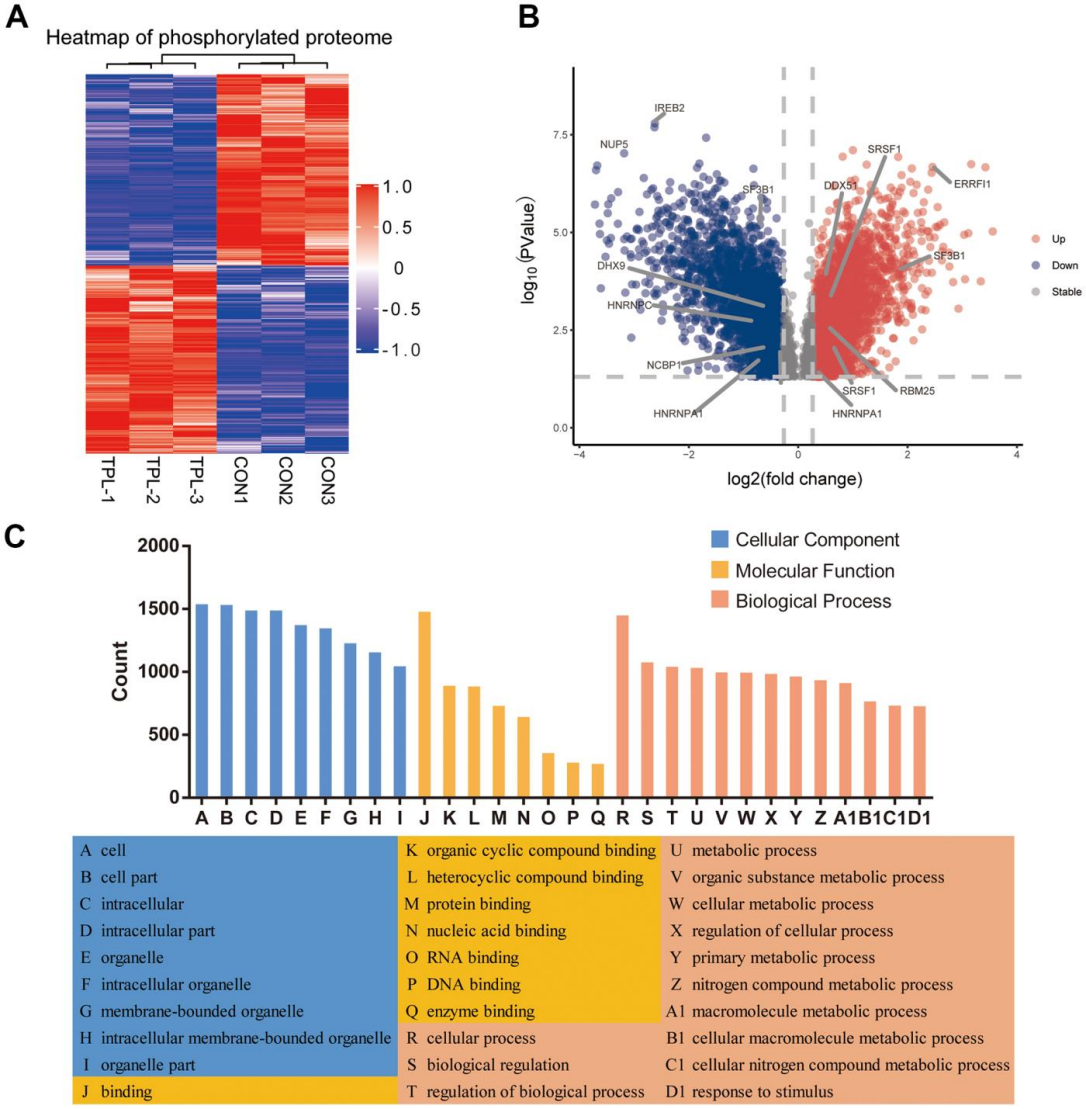
**Differential phosphorylation of the quantitative proteome and enrichment in Gene Ontology terms.** (**A**) Heatmap based on differential phosphorylation levels. (**B**) Volcano plot of the differences in phosphorylation levels. (**C**) Classification of differentially phosphorylated proteins based on Gene Ontology biological processes, cellular components and molecular functions.

A total of 3410 quantitative proteins were identified in the experimental group and the control group (Supplementary Figure 2B). Similarly, we identified 3110 proteins with higher phosphorylation levels and 3161 proteins with lower phosphorylation levels in triptolide-treated cells group than in the control group (Figure 4A). Besides, a total of 17,056 phosphosites were identified, of which 88.22% were serines, 11.33% were threonines, and 0.45% were tyrosines (Supplementary Figure 4). The R package “pheatmap” was used to draw a heatmap (Figure 4B), which shows the expression levels of the differentially expressed proteins at the phosphorylation site screened by the volcano map.

Analysis of differentially phosphorylated proteins showed enrichment of the following GO biological processes (Figure 4C and Supplementary Figure 5A–5C): cellular processes, biological regulation of biological processes, regulation of cellular processes, response to stimulus, cellular response to stress, and nucleic acid metabolism. The proteins were enriched in the following GO cellular components: nucleus, organelles, intracellular space, membrane−enclosed lumen, and nuclear lumen. Differentially phosphorylated proteins were enriched in the following GO molecular functions: binding, catalytic activity, heterocyclic compound binding, organic cyclic compound binding, nucleic acid binding, protein binding, RNA binding and cytoskeletal protein binding.

### Analysis of differentially phosphorylated proteins for enrichment in domains and KEGG pathways, protein-protein interactions and modules

**Figure 5:**
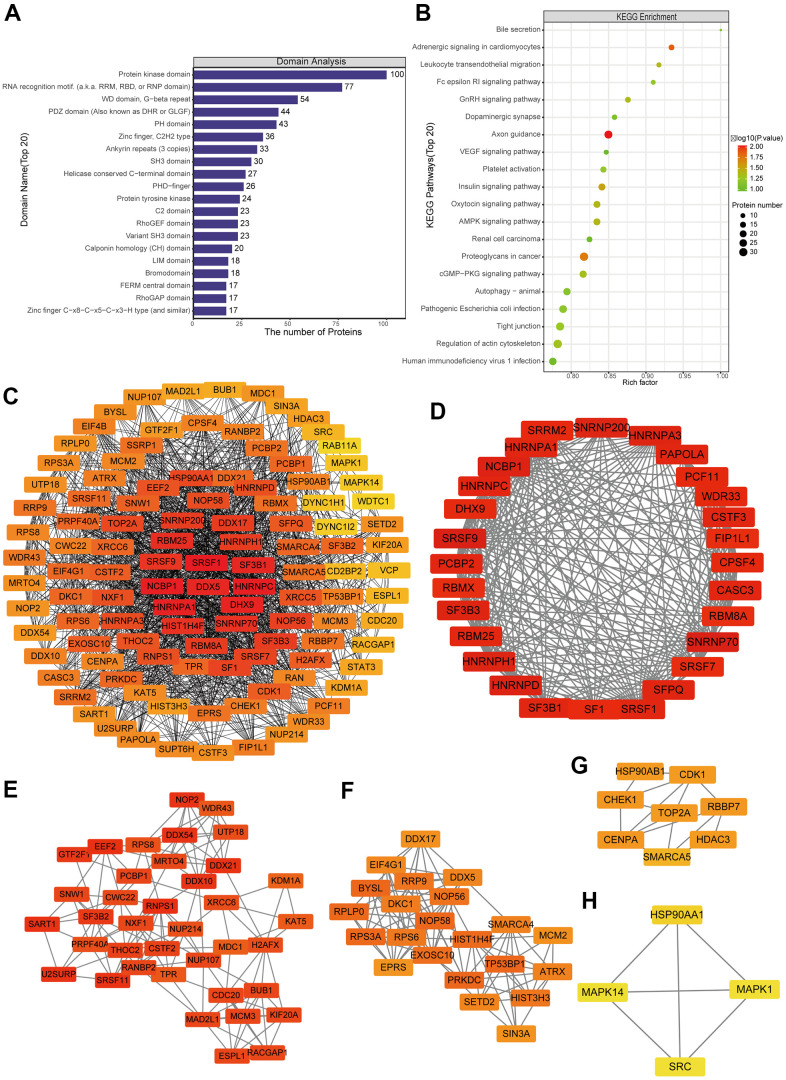
**Protein-protein interaction (PPI) network analyses of PDEPs were performed, and the four most significant modules were identified by the molecular complex detection (MCODE) algorithm.** (**A**) Enrichment of domains in differentially expressed proteins. (**B**) Enrichment of KEGG pathways in differentially expressed proteins. (**C**) Interaction network of differentially expressed proteins. (**D**–**H**) The five most significant MCODE modules.

Differentially phosphorylated proteins were enriched in several domains (Figure 5A and Supplementary Figure 5E): protein kinase, RNA recognition motif, WD, G−beta repeat, PDZ, LIM, PHD−finger, and KH. The proteins were enriched in the following KEGG pathways (Figure 5B and Supplementary Figure 5D): proteoglycans in cancer, human immunodeficiency virus 1 infection, regulation of actin cytoskeleton, tight junction, pathogenic *Escherichia coli* infection, animal autophagy, cGMP−PKG signaling, renal cell carcinoma, AMPK signaling, and axon guidance.

A network of potential interactions among differentially phosphorylated proteins (Figure 5C) led to the identification of five critical groups (Figure 5D–5H): MCODE 1 (MCODE score = 19.778), consisting of 28 nodes and 267 edges; MCODE 2 (score = 9.81), consisting of 22 nodes and 103 edges; MCODE 3 (score = 7.459), comprising 38 nodes and 138 edges; MCODE 4 (score = 4.286), comprising 8 nodes and 15 edges; and MCODE 5 (score = 4), comprising 4 nodes and 6 edges. The four classification methods in CytoHubba (Supplementary Table 2) converged on the following eight proteins as hub phosphorylated proteins: SRSF1, HNRNPC, NCBP1, HNRNPA1, DHX9, DDX5, RBM25 and SF3B1 (Supplementary Figure 6B).

### Motif analysis of the phosphosites

**Figure 6:**
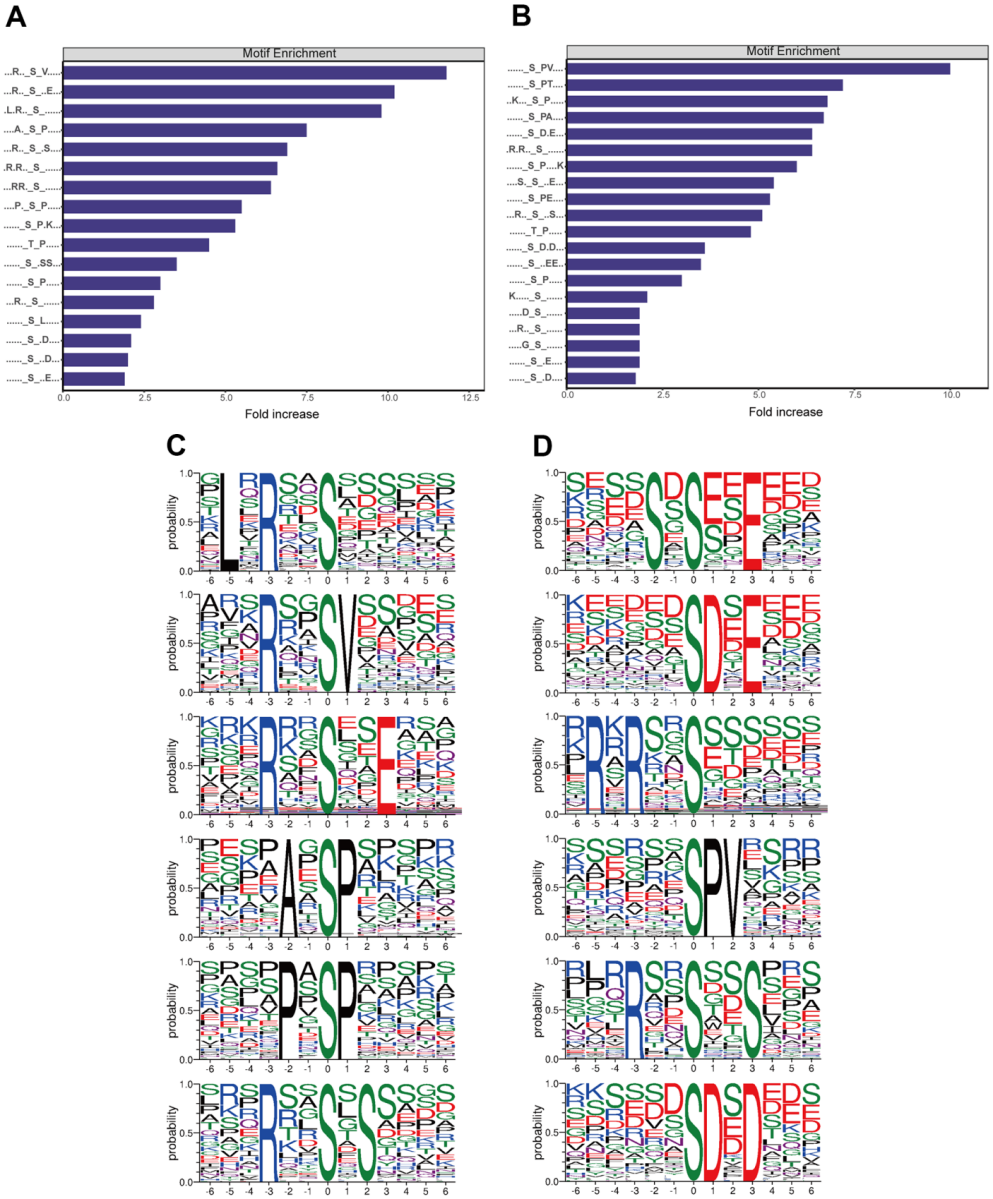
**Analysis of motifs differentially phosphorylated between CRC cultures treated with triptolide or vehicle.** (**A**) Motifs whose phosphorylation is upregulated by triptolide. (**B**) Motifs whose phosphorylation is downregulated by triptolide. (**C**) Ranking of the top six motifs upregulated by triptolide. (**D**) Ranking of the top six motifs downregulated by triptolide.

Among the protein sequences differentially phosphorylated between triptolide and control CRC cultures, we identified 50 conserved motifs in which a serine was phosphorylated and 7 conserved motifs in which a threonine was phosphorylated (Supplementary Table 2). Several of the motifs were upregulated by triptolide (Figure 6A), while other motifs were downregulated (Figure 6B). Based on motif score, we identified the top six hub motifs that were down- or upregulated (Figure 6C, 6D).

According to the Human Protein Reference Database (HPRD), 16 phosphorylation motifs have previously been verified as substrates of certain protein kinases, while 41 have not yet been linked to kinases (Supplementary Table 2). Particularly conserved motifs were [xpSxxxx_pS_PxxxxK] (motif 1), [xxpSxxx_pS_PxxxK] (motif 3), [xxpSxxx_pS_PpTxxx] (motif 5), [xxxRpSx_pS_xpSxxx] (motif 15) and [xxxxSx_pS_ExExxx] (motif 24). All these motifs scored > 40.00. Several motifs have previously been shown to be phosphorylated by casein kinase II [11–14]: [xxxxxx_S_xExxxx] (motif 8), [xxxxxx_S_DxExxx] (motif 16), [xxxxxx_S_EEExxx] (motif 25), [xxxxxx_S_xDxxxx] (motif 40), [xxxSxx_S_xxxxxx] (motif 50), [xxxxxx_T_xxExxx] (motif 55), and [xxxxSx_T_xxxxxx] (motif 56). Casein kinase II is upregulated in numerous cancers, and it has been proposed as a therapeutic target in CRC [15–17]. Meanwhile, elevated Casein kinase II activity play a role in transcriptional regulator of cell cycle and PI3K-promoting genes [18]. The motifs [xxxxxx_S_Pxxxxx] (motif 26) and [xxxxxx_T_Pxxxxx] (motif 54) are known to be phosphorylated by kinases containing a WW domain [19–21]. The motif [xLxRxx_S_xxxxxx] (motif 29), for its part, is phosphorylated by calmodulin-dependent protein kinase II [22], which may be a therapeutic target in cancer [23]. In this way, our findings identify several kinases that may help mediate the effects of triptolide against CRC.

### Verification with molecular docking

**Figure 7:**
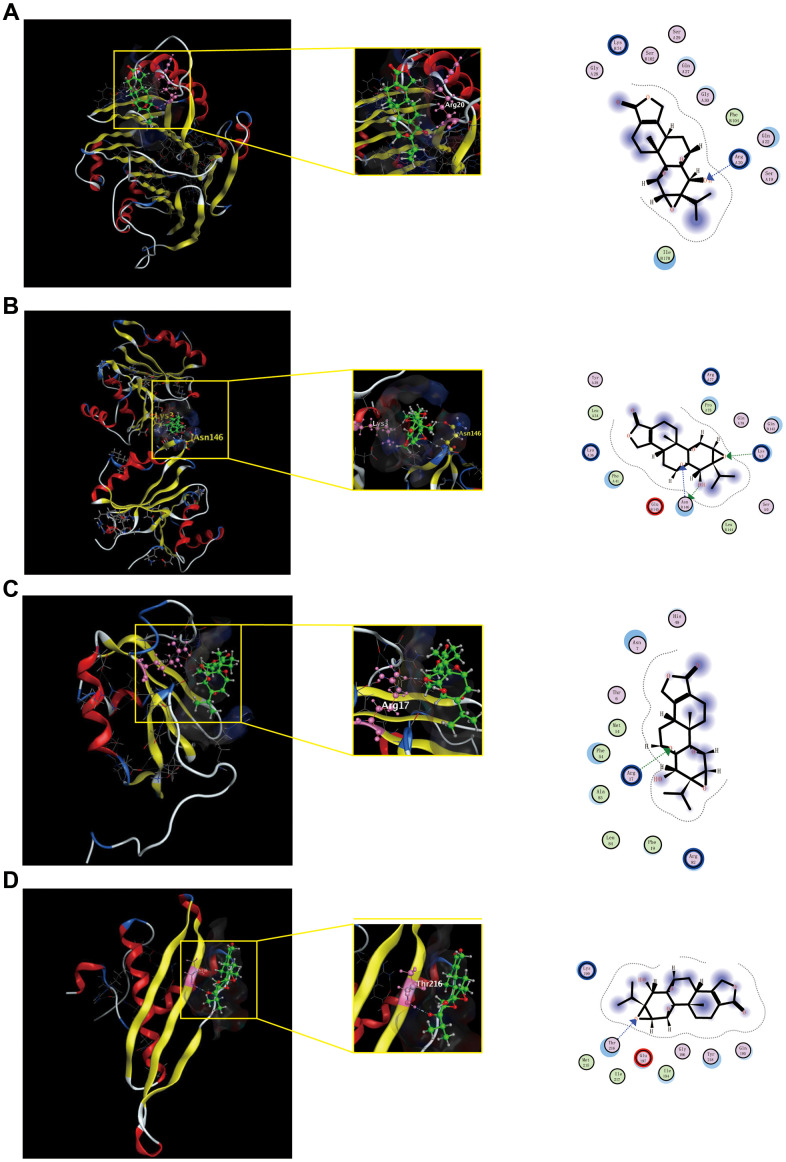
**Shows the binding interactions of triptolide with the CRC-related hub genes protein.** Triptolide binds to AMD1(**A**), IMP3(**B**), HNRNP(**C**) and DHX9(**D**). Ball and stick represent triptolide; cartoon represents a hub target.

To further validate potential targets in triptolide, we performed molecular docking with hub genes. Docking analysis successfully predicted binding energy (ΔGb), which were all negative and less than −5, between quercetin and the hub genes. The scores of triptolide-AMD1, -IMP3, -HNRNPC, -DHX9 was −5.7634, −6.1944, −5.5740 and −5.4239 kcal/ mol, respectively (Supplementary Table 3). Docked compounds showed hydrogen bonds in the active site. These selected compounds bind to the hub genes protein by interacting with different amino acid residues, such as Arg20, Lys 3, Asn146, Arg17 and Thr 216. Overall, molecular docking results indicated that triptolide had good binding activities to AMP1, IMP3, HNRNPC and DHX9, as shown in Figure 7.

## DISCUSSION

Globally CRC is the third most frequent cancer and the second most frequent cause of cancer-related deaths [24]. Triptolide has been reported to affect CRC in various ways, such as by arresting the cell cycle [4, 25] and decreasing vascular endothelial growth factor expression to inhibit migration [26]. Since CRC onset and progression likely involve complex interactions among many genes and proteins [27, 28], we did not focus here on specific proteins but instead examined the entire (phospho)proteomic landscape using liquid chromatography-tandem mass spectrometry [29]. We identified 559 proteins whose expression was downregulated and 403 proteins whose expression was upregulated by triptolide.

For example, we found that triptolide downregulated ZFP36L2, consistent with previous studies [30, 31]. In the case of pancreatic ductal adenocarcinoma, high expression of ZFP36L2 predicts shorter survival, and silencing it inhibits cancer cell aggressiveness [31]. We also found that triptolide downregulated AMD1, which is upregulated in many cancers and is associated with patient prognosis [32, 33]. Similarly, triptolide downregulated the RNA helicase DHX9, which is highly expressed in several cancers and is involved mainly in RNA splicing and processing, ribosome synthesis, as well as translation and transcription [34]. Triptolide downregulated the RNA-binding protein HNRNPC. This protein is upregulated in various cancers, and its inhibition slows cancer cell proliferation and tumor growth [35]. Our research highlights that triptolide can directly or indirectly phosphorylate HNRNPC and it is down regulated in triptolide treated group. Therefore, we attribute that triptolide may mediate the proliferation of tumor by HNRNPC. These indicates that triptolide plays a critical role in a variety of cellular processes, especially in cell growth, cell migration and immunoreactivity.

Many of the GO terms enriched in the proteins whose expression was altered by triptolide localized to the nucleus and were related to the ribosome. An important feature of cancer cells is increased ribosomal production and strong disruption of ribosome biogenesis [36, 37]. The production of functional ribosomes begins in the nucleolus [38–40], so this may be an important site of triptolide anticancer activity.

Triptolide downregulated hedgehog signaling, and it altered the phosphorylation of proteins involved in PI3K−Akt signaling and MAPK signaling. Hedgehog signaling has been linked to cancer, in particular for maintaining tumor-initiating/stem cells [41]. The pathway contributes to tumorigenesis and tumor growth through several mechanisms [42, 43], including processes affecting cell proliferation, survival and angiogenesis [44]. The pathway can be activated by TNF-α, KRAS–MAPK/ERK, and PI3K–Akt [45–47]. In fact, PI3K activates Akt to regulate hedgehog signaling during the specification of neuronal fate [48]. Our results suggest that triptolide acts partly through hedgehog and associated signaling pathways.

We found that triptolide downregulated the RNA-binding protein IMP3, which is required for ribosomal RNA processing and may predict prognosis in many cancers [49–51]. In breast cancer, IMP3 activates TAZ, a transcriptional co-activator of Hippo signaling that helps drive breast cancer stem cell function [49]. In prostate cancer, IMP3 is overexpressed, and it accelerates the cancer’s progression by increasing SMURF1-mediated PTEN ubiquitination, which in turn activates PI3K/AKT/mTOR signaling [50]. In CRC, IMP3 regulates MEKK1 to activate MEK1/ERK signaling, driving cancer progression [52]. Our results suggest that triptolide acts in part through IMP3 and associated pathways. Clinical therapeutic effect need to be further validated in controlled clinical trials.

Altogether, our analysis identifies several pathways through which triptolide may suppress CRC proliferation, including pathways involving IMP3/ PI3K/AKT/mTOR, Hedgehog/ PI3K/AKT and ZFP36L2, AMD1, DHX9 and HNRNPC. These results may help optimize the anticancer efficacy of triptolide as well as develop new druggable targets against CRC.

## MATERIALS AND METHODS

### Cell culture and treatment

The human colon carcinoma cell line HCT 116 was obtained from National Infrastructure of Cell Line Resource (Beijing, China). Cells were treated for 48 h with triptolide (40 ng/ml) dissolved in DMSO or DMSO vehicle. The medium for all cell culture was RPMI 1640 (Life Technologies, Shanghai, China) supplemented with 10% fetal bovine serum (FBS; Thermo Scientific, Shanghai, China). Cultures were incubated at 37° C in an atmosphere of 5% CO**_2_**.

### Protein extraction and preparation

HCT116 cells were cultured to 70% confluence, then lysed using a buffer containing 100 mM Tris-HCl (pH 7.6), 4% SDS, 1 mM DTT. Protein concentration were quantified using the BCA assay (Bio-Rad, Hercules, California, USA). The protein solution was sequentially diluted (5 mmol/L dithiothreitol for 30 min at 56° C) and alkylated with 11 mmol/L iodoacetamide for 15 min. These procedures were performed in darkness at room temperature. Then, the assembled protein sample was diluted to a urea concentration of less than 2 mol/L. Finally, trypsin was added to initiate overnight digestion (the ratio of trypsin to the protein mass ratio was 1:50) at 37° C and a subsequent 4 h digestion (the ratio of trypsin to protein mass was 1:100). The resulting peptides were desalted on a Empore™ SPE C18 cartridge (standard density, 7 mm inner bed diameter, 3 ml volume; Sigma, Shanghai, China). The eluted peptides were concentrated by vacuum centrifugation and reconstituted in 40 μl of 0.1% (v/v) formic acid.

### Tandem mass tagging and enrichment of phosphopeptides

Tryptic peptide mixtures were labeled with TMT Reagent (Thermo Fisher Scientific) according to the manufacturer’s instructions. Three independent cultures of untreated HCT116 were tagged (tags 126, 127 and 128), as well as three independent cultures of triptolide-treated HCT116 cells (tags 129, 130 and 131). Peptide mixtures were enriched for phosphorylated peptides using the High-Select™ Fe-NTA Kit (Thermo Scientific) according to the manufacturer’s instructions. The resulting phosphopeptide mixtures were lyophilized, then resuspended in 20 μL of 0.1% (v/v) formic acid.

### Liquid chromatography-tandem mass spectrometry

Total peptide and phosphopeptide-enriched samples were loaded onto an Acclaim PepMap100 nanoViper C18 reverse-phase trap column (Thermo Scientific; dimensions, 100 μm x 2 cm) connected to an Easy C18 reverse-phase analytical column (Thermo Scientific; inner diameter, 75 μm; length, 10 cm; resin diameter, 3 μm) in buffer A (0.1% formic acid). Peptides were separated using a linear gradient of buffer B (84% acetonitrile, 0.1% formic acid) at a flow rate of 300 nl/min.

The separated peptides were then subjected to tandem mass spectrometry on a Q Exactive mass spectrometer (Thermo Scientific) for 60-90 min, operated in positive ion mode. Data were acquired using a data-dependent top10 method that dynamically selected the most abundant precursor ions from the survey scan (300–1800 m/z) for Higher energy Collision Induced Dissociation (HCD) fragmentation. The system was operated in peptide recognition mode, and the following device parameters were used: automatic gain control target, 3e6; maximum injection time, 10 ms; dynamic exclusion duration, 40.0 s; survey scan resolution, 70,000 at m/z 200; HCD spectrum resolution, 17,500 at m/z 200; isolation width, 2 m/z; normalized collision energy, 30 eV; and underfill ratio (minimum percentage of the target value likely to be reached at maximum fill time), 0.1%.

### Database search

The resulting MS/MS data were processed using the MASCOT engine (Matrix Science, London, UK; version 2.2) embedded into Proteome Discoverer 2.4. The data were searched against the database “Homo_sapiens_194324” and against a library of common protein contaminants (for filtering out contaminant proteins), and an anti-database was added to assess the false discovery rate (FDR) due to random matches. The following system parameters were applied: restriction enzyme digestion method, trypsin/P; number of missed cleavage sites, 2; peptide mass tolerance, ± 20.0 ppm; fragment mass tolerance, 0.1 Da; fixed modification, carbamidomethyl (C); variable modifications, “Oxidation (M)”, “Phospho(ST)”, “Phosp (Y)”; and FDR, 1%.

Only proteins whose levels differed > 2-fold or < 0.83-fold between cultures treated with triptolide or vehicle (in association with p < 0.05) were considered in subsequent bioinformatics analyses. A similar criterion was applied to select phosphorylation sites in the proteome.

### Bioinformatic analyses

Differentially expressed proteins were searched against the NCBI BLAST+ database (ncbi-blast-2.2.28+-win32.exe) and homologous sequences were identified using InterProScan. Potential functions of the proteins were explored using Gene Ontology (GO) terms and annotated using Blast2GO (https://www.blast2go.com/) according to GO biological processes, cellular components and molecular functions.

After annotation, proteins were mapped to Kyoto Encyclopedia of Genes and Genomes (KEGG) pathways (http://www.genome.jp/kegg/). Their subcellular localizations were predicted using CELLO (http://cello.life.nctu.edu.tw). In addition, the InterPro (providing resources for functional analysis of protein sequence family classification, prediction of structural domains and special sites) database was used to analyze the enrichment of functional domains of differentially expressed proteins. Enrichment of a given differentially expressed protein or protein domain was defined as p < 0.05 in a two-tailed Fisher’s exact test. We examined enrichment in terms of GO terms, KEGG and domains. Categories that contained at least one enriched cluster and that were associated with p < 0.05 were considered significant.

The STRING database (version 10.5) was used to create a protein-protein interaction network, and interactions with a confidence score > 0.7 were considered probable. Finally, we integrated databases and protein-protein interaction network, then explored densely connected regions using MCODE and Cytohubba.

Phosphorylation motifs were analyzed using MeMe (http://meme-suite.org/index.htm). We extracted amino acid sequences containing the phosphorylated residue as well as six residues upstream and six downstream. Only when the minimum number of occurrences was set to 20 and the statistical test P value is less than 0.000001, the characteristic sequence form is considered to be a motif of the modified peptide. Finally, we estimate the molecular binding capacities of the compounds with the target proteins. The structures of triptolide were downloaded from the TCMSP database. Then, the downloaded structures were converted to three dimensional (3D) structures, and the energy of them was minimized through the Molecular Operating Environment (MOE) 2019.10 software. Molecular docking analysis was conducted for comparing the combined action between the compounds and the crystal structures of AMD1 (PDB ID: 3DZ7), IMP3 (PDB ID:6FQR), HNRNPC (PDB ID: 2MZ1), DHX9 (PDB ID: 3VYX) using MOE. For each molecular compounds, a number of placements called poses. Among the placement of the compounds, the best pose with the lowest binding energy (ΔGb) was selected as the output result.

## Supplementary Material

Supplementary Figures

Supplementary Tables
