# Supplementary material for: Mechanisms of action of triptolide against colorectal cancer: insights from proteomic and phosphoproteomic analyses
Source: Aging (Albany NY). 2022 Apr 2;14(7):3084–104. doi: 10.18632/aging.203992 (PMC9037262; doi:10.18632/aging.203992)
Supplement: Supplementary Figures [file aging-14-203992-s001.pdf]

SUPPLEMENTARY FIGURES

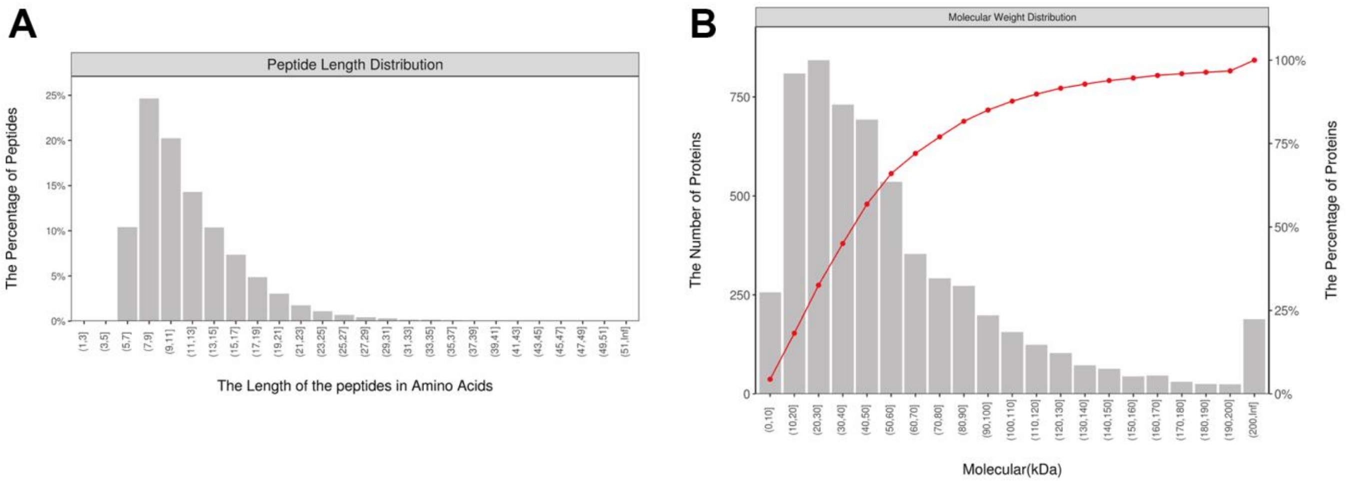

Supplementary Figure 1. (**A**) Distribution of peptide lengths identified by mass spectrometry. (**B**) Distribution of molecular weights of all proteins identified.

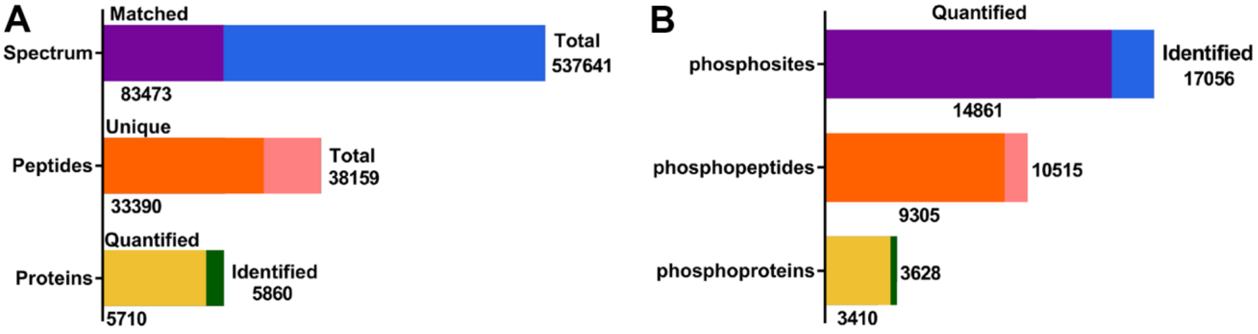

Supplementary Figure 2. (**A**) Results of proteome quantitation. (**B**) Results of phosphoproteome quantitation.

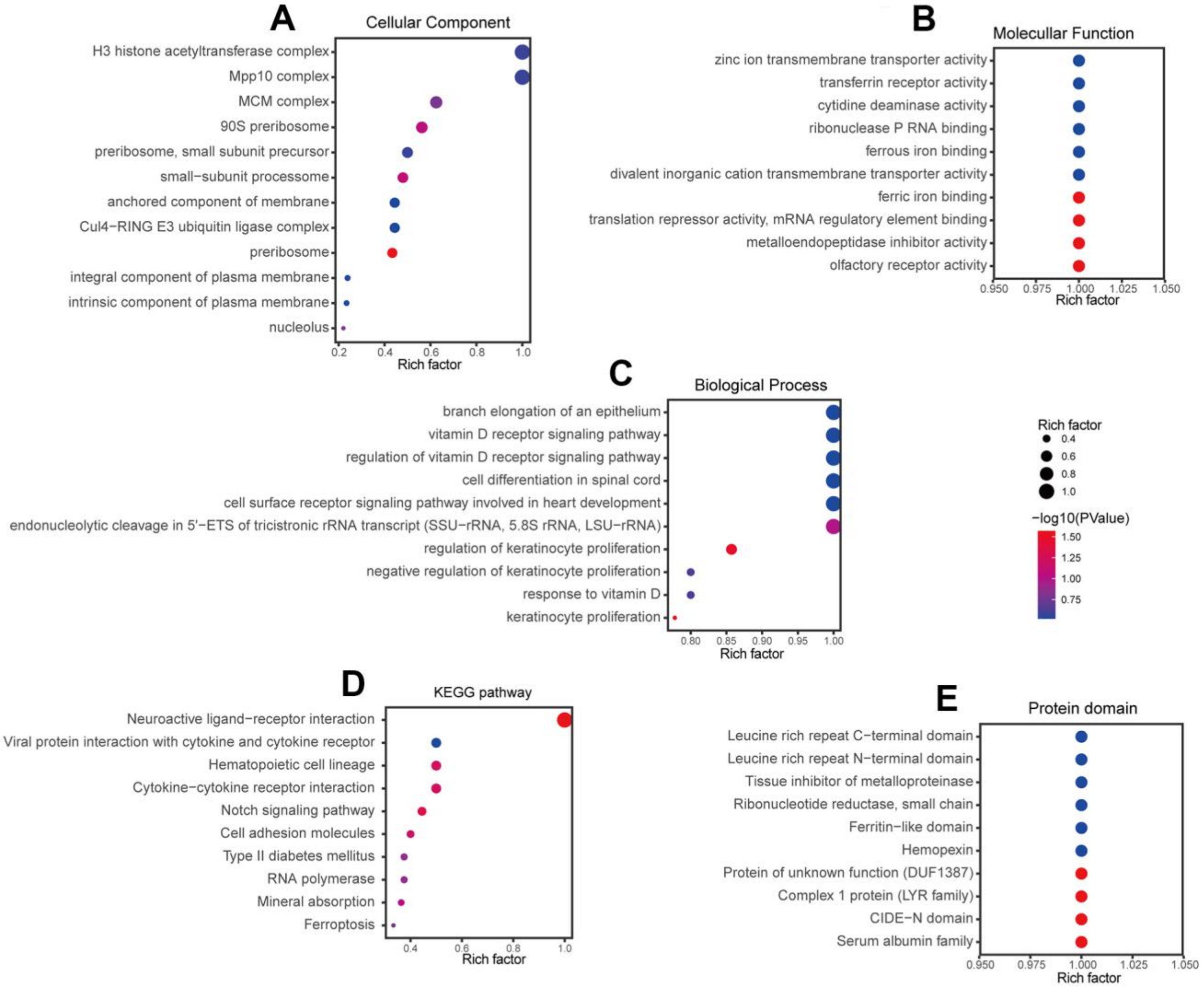

Supplementary Figure 3. (**A**) Clustering of differentially expressed proteins based on enrichment in Gene Ontology (GO) cellular components. (**B**) Clustering of differentially expressed proteins based on enrichment in GO molecular functions. (**C**) Clustering of differentially expressed proteins based on enrichment in GO biological processes. (**D**) Clustering of differentially expressed proteins based on enrichment in Kyoto Encyclopedia of Genes and Genomes (KEGG) pathways. (**E**) Clustering of differentially expressed proteins based on protein domains.

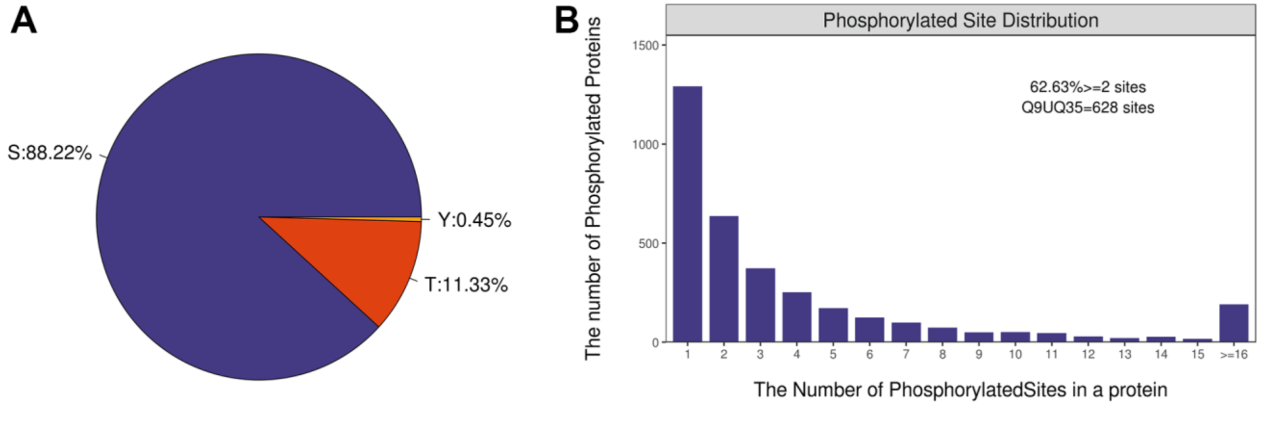

Supplementary Figure 4. (**A**) Distribution of serine (S), threonine (T) and tyrosine (Y) phosphorylation among all phosphoproteins identified by mass spectrometry. (**B**) Distribution of phosphoproteins based on number of phosphorylation sites per protein.

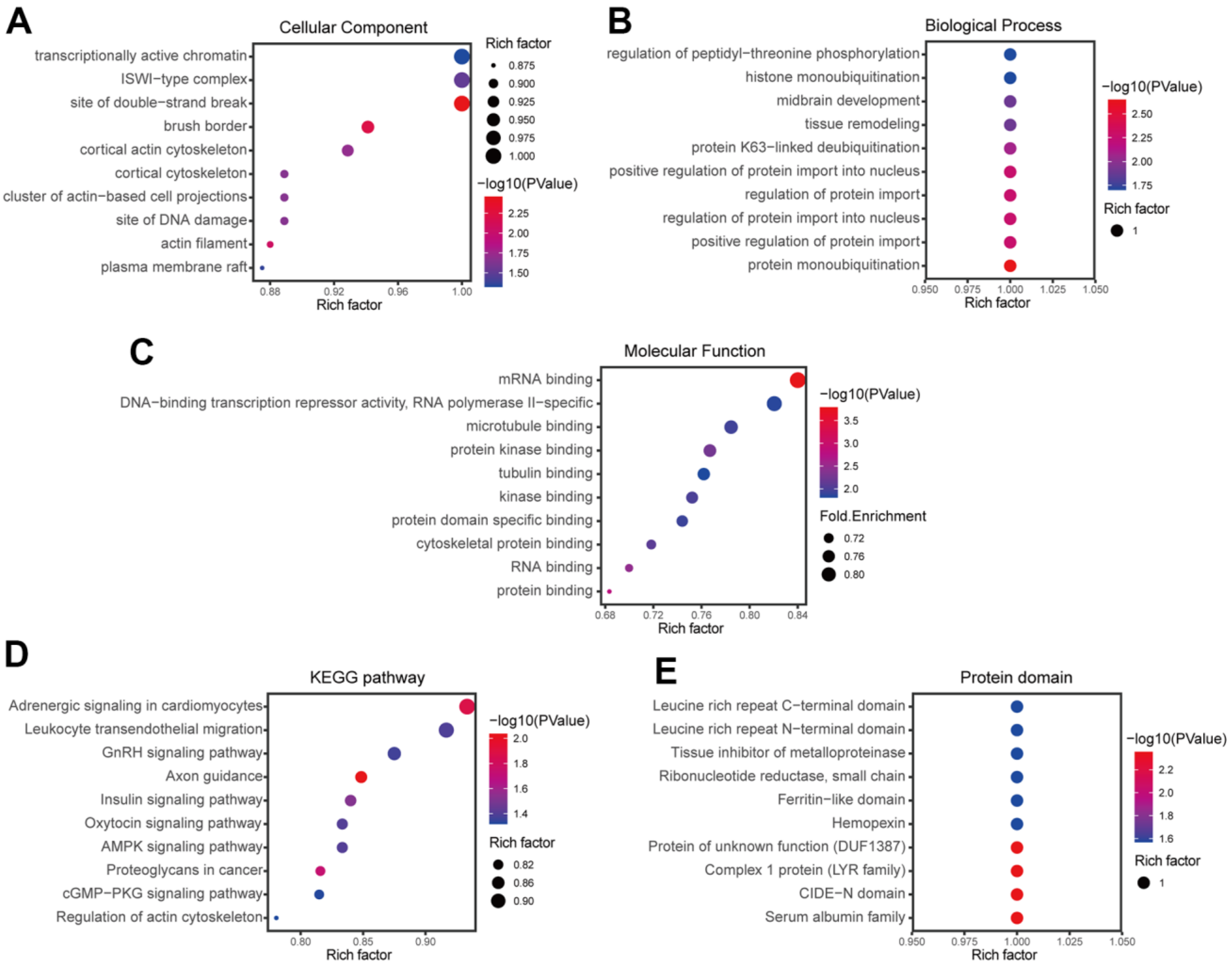

Supplementary Figure 5. (**A**) Clustering of differentially phosphorylated proteins based on enrichment in Gene Ontology (GO) cellular components. (**B**) Clustering of differentially phosphorylated proteins based on enrichment in Gene Ontology (GO) biological processes. (**C**) Clustering of differentially phosphorylated proteins based on enrichment in Gene Ontology (GO) molecular functions. (**D**) Clustering of differentially phosphorylated proteins based on enrichment in Kyoto Encyclopedia of Genes and Genomes (KEGG) pathways. (**E**) Clustering of differentially phosphorylated proteins based on enrichment in protein domains.

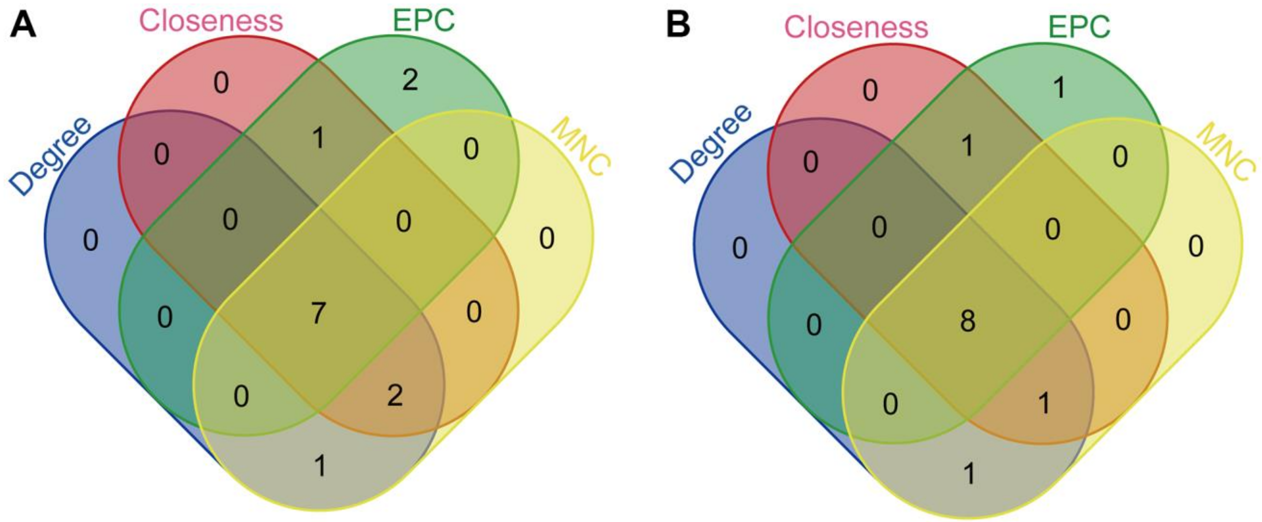

Supplementary Figure 6. (**A**) Venn diagram of differentially expressed proteins screened by four classification methods in order to identify hub proteins. (**B**) Venn diagram of differentially phosphorylated proteins screened by four classification methods in order to identify hub phosphorylated proteins.
