# Supplementary material for: Mechanisms of action of triptolide against colorectal cancer: insights from proteomic and phosphoproteomic analyses
Source: Aging (Albany NY). 2022 Apr 2;14(7):3084–104. doi: 10.18632/aging.203992 (PMC9037262; doi:10.18632/aging.203992)
Supplement: Supplementary Tables [file aging-14-203992-s002.pdf]

SUPPLEMENTARY TABLES

Supplementary Table 1. The top ten proteins screened by four classification methods.

Degree	Closeness	EPC	MNC
IMP3	IMP3	IMP3	IMP3
BYSL	PDCD11	BYSL	BYSL
PDCD11	BYSL	PDCD11	PDCD11
PNO1	PNO1	RRS1	PNO1
NSA2	NSA2	RPF2	NSA2
MPHOSPH10	MPHOSPH10	BMS1	MPHOSPH10
RPF2	RPF2	NOC4L	RPF2
RSL1D1	RRS1	NSA2	RSL1D1
RBM28	NOC4L	PNO1	RBM28
RRS1	RSL1D1	KIAA0020	RRS1

Supplementary Table 2. The top ten phosphorylation modified proteins screened by four classification methods.

Degree	Closeness	EPC	MNC
SRSF1	SRSF1	SRSF1	SRSF1
NCBP1	HNRNPC	HNRNPC	NCBP1
HNRNPC	NCBP1	HNRNPA1	HNRNPC
HNRNPA1	HNRNPA1	NCBP1	HNRNPA1
DHX9	DHX9	DHX9	DHX9
SF3B1	SF3B1	SF3B1	SF3B1
DDX5	DDX5	RBM25	DDX5
RBM25	HIST1H4F	SRSF9	RBM25
SNRNP70	RBM25	DDX5	SNRNP70
HIST1H4F	SRSF9	SF3B3	HIST1H4F

Supplementary Table 3. Interaction of hub genes with triptolide.

Gene name	PDB ID	Herbs	ΔGb	H-Bonds
				Type amino acid
AMD1	3DZ7	Triptolide	-5.2803--5.7650	H-donor Arg *A*-20
IMP3	6FQR	Triptolide	-5.6453--6.1944	H-donor Lys *A*-3
				H-donor Asn *B*-146
				H-acceptor Asn *B*-146
HNRNPC	2MZ1	Triptolide	-5.0554--5.8036	H-donor Arg 17
DHX9	3VYX	Triptolide	-5.3092--5.4239	H-donor Thr 216
